# Autoimmune hemolytic anemia: current knowledge and perspectives

**DOI:** 10.1186/s12979-020-00208-7

**Published:** 2020-11-20

**Authors:** Sylwia Sulimiera Michalak, Anna Olewicz-Gawlik, Joanna Rupa-Matysek, Edyta Wolny-Rokicka, Elżbieta Nowakowska, Lidia Gil

**Affiliations:** 1 Department of Pharmacology and Toxicology Institute of Health Sciences, Collegium Medicum, University of Zielona Gora, Zielona Góra, Poland; 2 Department of Anatomy and Histology Institute of Health Sciences, Collegium Medicum, University of Zielona Gora, Zielona Góra, Poland; 3 Department of Infectious Diseases, Hepatology and Acquired Immune Deficiencies, Poznan University of Medical Sciences, Poznan, Poland; 4 Department of Immunology, Poznan University of Medical Sciences, Poznan, Poland; 5 Department of Hematology and Bone Marrow Transplantation, Poznan University of Medical Sciences, Poznań, Poland; 6 Department of Radiotherapy, Multidisciplinary Hospital, Gorzów Wielkopolski, Poland

**Keywords:** Autoimmune hemolytic anemia, Cold agglutinin disease, Pathogenesis, Microvesicles, Shear stress, Treatment

## Abstract

Autoimmune hemolytic anemia (AIHA) is an acquired, heterogeneous group of diseases which includes warm AIHA, cold agglutinin disease (CAD), mixed AIHA, paroxysmal cold hemoglobinuria and atypical AIHA. Currently CAD is defined as a chronic, clonal lymphoproliferative disorder, while the presence of cold agglutinins underlying other diseases is known as cold agglutinin syndrome. AIHA is mediated by autoantibodies directed against red blood cells (RBCs) causing premature erythrocyte destruction. The pathogenesis of AIHA is complex and still not fully understood. Recent studies indicate the involvement of T and B cell dysregulation, reduced CD4+ and CD25+ Tregs, increased clonal expansions of CD8 + T cells, imbalance of Th17/Tregs and Tfh/Tfr, and impaired lymphocyte apoptosis. Changes in some RBC membrane structures, under the influence of mechanical stimuli or oxidative stress, may promote autohemolysis. The clinical presentation and treatment of AIHA are influenced by many factors, including the type of AIHA, degree of hemolysis, underlying diseases, presence of concomitant comorbidities, bone marrow compensatory abilities and the presence of fibrosis and dyserthropoiesis. The main treatment for AIHA is based on the inhibition of autoantibody production by mono- or combination therapy using GKS and/or rituximab and, rarely, immunosuppressive drugs or immunomodulators. Reduction of erythrocyte destruction via splenectomy is currently the third line of treatment for warm AIHA. Supportive treatment including vitamin supplementation, recombinant erythropoietin, thrombosis prophylaxis and the prevention and treatment of infections is essential. New groups of drugs that inhibit immune responses at various levels are being developed intensively, including inhibition of antibody-mediated RBCs phagocytosis, inhibition of B cell and plasma cell frequency and activity, inhibition of IgG recycling, immunomodulation of T lymphocytes function, and complement cascade inhibition. Recent studies have brought about changes in classification and progress in understanding the pathogenesis and treatment of AIHA, although there are still many issues to be resolved, particularly concerning the impact of age-associated changes to immunity.

## Background

Autoimmune hemolytic anemia (AIHA) is characterized by hemolysis, i.e. the breakdown of red blood cells (RBCs) which occurs with autoantibodies and/or complement, together with activated macrophages, T-lymphocytes and cytokines all contributing to the process. All these immune parameters change with age, and immunosenescence is one of the pathomechanisms that has been associated with autoimmunity [1]. A positive direct antiglobulin test (DAT) confirms the presence of immunoglobulins (most often of the IgG class, sometimes IgM and IgA and/or complement - usually C3d) attached to erythrocytes [2].

The serological types of AIHA include warm autoimmune hemolytic anemia (wAIHA), cold agglutinin disease (CAD), mixed type AIHA (mixed AIHA) and paroxysmal cold hemoglobinuria (PCH). Recently, an atypical form of AIHA with DAT negative and the presence of IgA and warm IgM has been distinguished [2]. Primary CAD, according to the current modified definition, includes cases with low grade lymphoproliferative disorder (LPD) or unclassified B-cell lymphoproliferation in bone marrow [3]. The presence of cold agglutinins in the course of other diseases (especially SLE, Mycoplasma pneumoniae, Epstein-Barr infection or aggressive lymphoma) is defined as cold agglutinin syndrome (CAS) [4]. AIHA can be primary, when the underlying disease has not been demonstrated, or secondary. In approximately 50% of cases, the primary form of AIHA is diagnosed, while in other cases the autoantibodies are related to autoimmune diseases, lymphoproliferative diseases, infections (also SARS-CoV-2 infection), solid tumors or solid organ transplantation (Table 1) [3, 5, 12, 13]. The condition is also seen in patients after allogeneic stem cell transplantation (HSCT) with increasing incidence (reaching 2–6%), severe course and a high mortality rate [14–17]. Hemolysis which occurs after drugs is known as drug-induced immune hemolytic anemia (DIIHA), and is currently classified as a secondary form of AIHA [3].

**Table 1:** Most common secondary conditions associated with different types of AIHA [5–7]

Type of AIHA	Etiology
Warm AIHA	Hematologic disorders and lymphoproliferative diseases (CLL, Hodgkin’s and non-Hodgkin’s lymphoma)
	Solid malignancy (thymoma, ovarian or prostate carcinoma)
	Autoimmune diseases (SLE, Sjögren syndrome, sytemic sclerosis, rheumatoid arthritis, colitis ulcerosa, PBC)
	Viral infections (HCV, HIV, VZV, CMV, SARS-CoV-2)
	Bacterial infections (tuberculosisis, pneumococcal infections)
	Leishmania parasites
	Bone marrow or solid-organ transplantation
	Primary immune deficiency syndromes (CVID, ALPS)
	Sarcoidosis
CAD	Lymphoproliferative diseases (Waldenström macroglobulinemia, non-Hodgkin’s lymphoma)
	Solid malignancy
	Infections (parvovirus B19, Mycoplasma sp., EBV, adenovirus, influenza virus, VZV infections and syphilis)
	Autoimmune disease
	Post-allogeneic HSCT
PCH	Bacterial infections (Mycoplasma pneumoniae, *Haemophilus influenzae*, *Escherichia coli* infections and syphilis)
	Viral infections (adenovirus, influenza A virus, VZV infection; mumps, measles)
	Myeloproliferative disorders
Mixed AIHA	Lymphoma
	SLE
	Infection
DIIHA	Antibiotics (cephalosporins, beta-lactamase inhibitors, cotrimoxazole)
	Antiviral drugs: HAART
	Anti-PD-1 monoclonal antibodies (nivolumab, pembrolizumab)
	Chemotherapy (carboplatin, oxaliplatin)
	Non-steroidal anti-inflammatory drugs (diclofenac)
	Others: methyldopa

*AIHA* autoimmune haemolytic anaemia, *ALPS* autoimmune lymphoproliferative syndrome, *anti-PD-1* anti programmed death-1, *CAD* cold agglutinin disease, *CLL* chronic lymphocytic leukaemia, *CMV* cytomegalovirus, *CVID* common variable immunodeficiency, *DIIHA* drug-induced immune hemolytic anaemia, *EBV* epstein-barr virus, *HAART* highly active antiretroviral therapy, *HCV* hepatitis C, *HIV* human immunodeficiency virus, *HSCT* haematopoietic stem cell transplantation, *PBC* primary biliary cirrhosis, *PCH* paroxysmal cold haemoglobinuria, *SLE* systematic lupus erytremathosus, *VZV* varicella zoster virus

Recently, in new data regarding the pathogenesis and AIHA, treatment options have been discussed. Therefore, this review is a summary of the current knowledge about this heterogeneous and still not fully understood disease, and how its characteristics may differ depending on the immunological status of older adults with AIHA.

## Main text

### Epidemiology and risk factors for AIHA development

It is currently estimated that the incidence of AIHA is 1.77 cases per 100,000 per year [18], of which wAIHA is the most common form and accounts for about 2/3 of cases [19]. CAD is the second most common, accounting for approximately 15–20% of AIHA cases [20]. CAD usually occurs in people > 50 years of age, most often in the 7th and 8th decades of life [21, 22]. PCH is a rare disease which mostly affects children [23]. It is extremely rare in adults and is often associated with infections in this age group [24]. The risk of AIHA increases with age, in wAIHA the risk is 5 times higher in the 7th decade of life compared to the fourth decade [21]. The main reason for this age dependency could be immunosenescence [25] or epigenetic abnormalities accumulated in hematopoietic cells with aging [26]. The aging processes as well as numerous comorbidities increase the probability and severity of oxidative stress and eryptosis, i.e. erythrocyte cell membrane changes leading to RBC senescence and premature death [27, 28]. Genetic background, immunodeficiency, autoimmune disease, infections, medication - especially novel anti-cancer drugs, neoplasia - especially CLL/NHL, and transplants have all been suggested as important risk factors for AIHA development [29]. The clinical course of AIHA can vary from mild to severe and life-threatening forms. The course of AIHA may be chronic or recurrent, and, very rarely can be episodic. It is estimated that the mortality in AIHA is about 10% [17, 30–32].

### Pathogenesis of AIHA

It is generally thought that autoimmunity is a result of the interaction of genetic predisposition and environmental factors. All components of the immune system, i.e. autoantibodies, cytokines, the complement system, phagocytes, B and T lymphocytes including cytotoxic CD8 + T cells and CD4 + T regulatory cells (Tregs), and NK cells are important players in the pathogenesis of AIHA, and all change with age. Similar to other autoimmune disorders, the development of AIHA is associated with dysregulation of the central and peripheral self-tolerance and the presence of autoreactive T and B cells [33, 34]. Naturally occurring CD4+ and CD25+ Tregs contribute to immunologic self-tolerance by suppressing potentially autoreactive T cells. A study on a murine model of AIHA showed that defective suppressive activity of CD4+ and CD25+ Tregs may be essential for the induction of autoantibodies against RBC and the maintenance of AIHA [35]. On the other hand, the findings of Richards et al. based on a study of other murine models of AIHA suggested that Tregs are not required for the prevention of RBC autoimmunity [36]. Howi et al. reviewed the current findings of existing animal models of AIHA and pointed to oxidative stress as a risk factor for AIHA and the reticulocytes as a target for pathogenic autoantibodies [37]. In particular, it was demonstrated in one model that reticulocytes had increased autoantibodies on their surface, produced more reactive oxygen species (ROS), and were preferentially cleared from the circulation [38]. The same group of researchers showed that anti-RBC pathogenic autoantibodies preferentially bound to reticulocytes and induced phosphatidylserine expression [39]. These results may thus explain why, in a subset of AIHA cases, reticulocytopenia is observed. Further findings from animal models showed that peripheral tolerance mechanisms may be more critical than thymic central tolerance [37].

T helper type 17 (Th17) cells and Treg cells share a common precursor cell (the naive CD4+ T cell), but play different roles in the immune response: Th17 cells are engaged in the development of inflammation and autoimmunity, whereas Treg cells inhibit these phenomena and maintain immune homeostasis. Xu et al. observed an increased number of Th17 cells in patients with AIHA, and the frequency of Th17 was closely related to the disease activity [40]. Furthermore, their results showed a close correlation between interleukin 17 (IL17) and the disease activity of patients with AIHA. They also confirmed the findings that Th17 cells can contribute to the development of AIHA [40]. It was demonstrated that the immune dysregulation present in AIHA may be associated with specific cytokine gene polymorphisms, which may then result in a Th17/Tregs imbalance [41–43]. Recent research into the AIHA mouse model has demonstrated the role of T follicular helper cells (Tfh) and T follicular regulatory cells (Tfr) in participating in B cell differentiation and regulation of anti-RBC antibody production [44]. The results of a recent study revealed that AIHA is also associated with the presence of clonal expansions of CD8+ T cells, yet the immune clones persisted during remission of AIHA and did not correlate with disease severity, duration, nor hemoglobin level, and were probably induced and accumulated during the autoimmune process [45].

About half of the cases of warm AIHA are recognized as secondary to underlying diseases, among others, infections. Molecular mimicry between self-antigens and pathogen antigens has been highlighted as one of the potential mechanisms by which pathogens could be involved in the induction and progression of AIHA [33, 34, 46]. It has been shown that a human immunodeficiency virus (HIV) infection may induce production of autoantibodies, owing to molecular mimicry [47]. Moreover, HIV infection is an independent risk factor for AIHA and increases the incidence of AIHA more than 20-fold [8, 48]. In the case of HIV infection, as well as in other immunodeficiencies (e.g. common variable immunodeficiency - CVID and autoimmune lymphoproliferative syndrome - ALPS), loss of immunological tolerance is essential [49–51].

There is also increasing evidence on the role of reactive oxygen species (ROS) in AIHA pathogenesis [34]. Iuchi et al. showed that increased oxidative stress in RBC caused by cytoplasmic Cu-Zn superoxide dismutase (SOD1) deficiency is associated with anemia and triggers autoantibody production [52]. In SOD1-deficient mice an elevation of the ROS levels in RBCs, oxidation of RBC components, and augmented production of autoantibodies in RBCs have been observed, with lipid peroxidation products, such as 4-hydroxy 2-nonenal and acrolein, present as epitopes for autoantibodies on RBC membranes [53]. Moreover, increased autoantibodies against RBCs correlated with elevated levels of ROS in these cells [34]. Additionally, animal AIHA models revealed that transgenic overexpression of human SOD1 in erythroid cells extended the life of mice and ameliorated AIHA symptoms [54], while antioxidants such as N-acetyl cysteine suppressed autoantibody production, supporting the oxidative stress theory of AIHA [34]. In addition, plasma-free heme, a breakdown product of hemoglobin released in the course of hemolysis, induces the formation of neutrophil extracellular traps (NET) through ROS signaling, thus affecting the regulation of immune cell function [55].

**Fig. 1:**
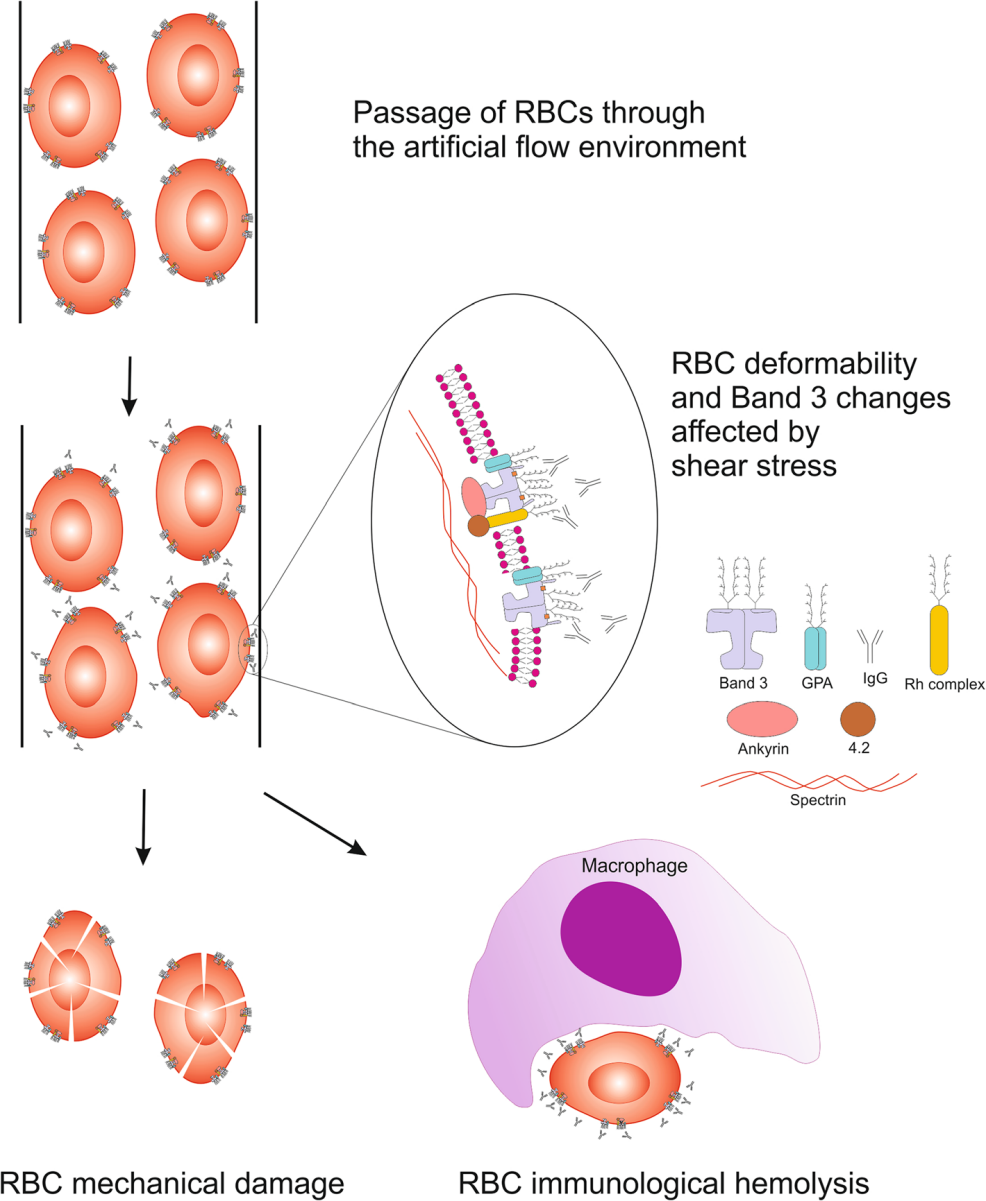
A schematic visualization of RBCs exposed to shear stress

A study by Buerck et al. pointed out the importance of shear stress in the induction and progression of the autoimmune response and accelerated senescence of RBC [56]. Their results showed increased binding of IgG to the RBC membrane exposed to high shear stress, indicating conformational changes in the RBC membrane protein, most probably the senescent antigen of band 3, thus exposing epitopes to naturally occurring antibodies (Fig. 1).

**Fig. 2:**
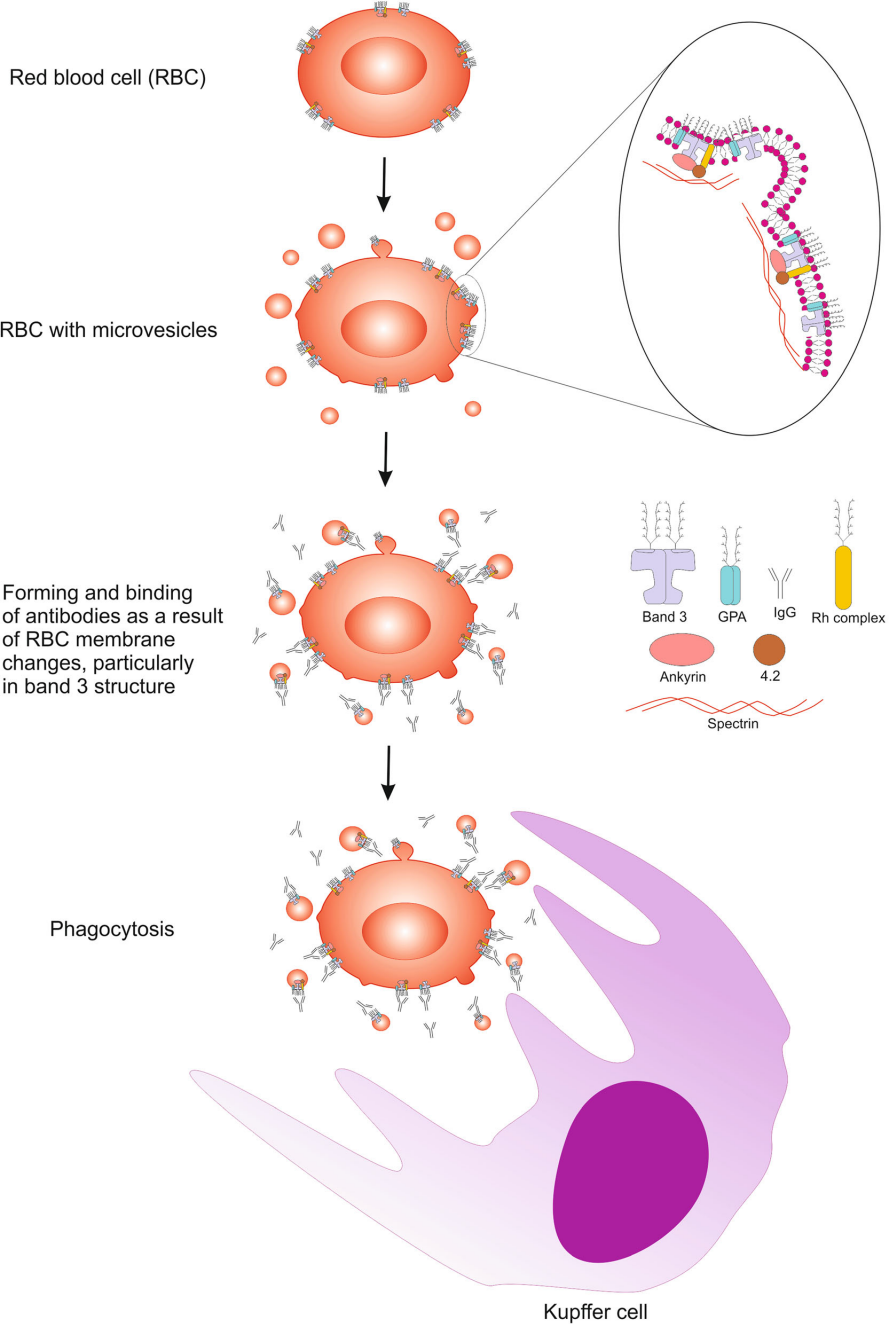
A schematic visualization of erythrocyte senescence and microvesicle generation

The microvesicles released from stored RBCs contain lipid raft proteins and oxidized signaling components commonly associated with the senescence of RBCs, the vesiculation contributes to irreversible membrane changes and activates an immune response [57, 58] (Fig. 2). Current observations suggest that erythrocyte-derived extracellular vesicles (EVs) from stored RBC units have immunomodulatory properties, including B lymphocyte vitality, plasma cell differentiation, and antibody production [59], and thus could influence the course of AIHA in patients receiving blood transfusions.

The genetic burden of AIHA has also been extensively investigated. In 1995 Michaux et al. found that trisomy 3 is a consistent chromosome change in CAD preceding lymphoproliferative malignancies [60]. Next-generation sequencing of bone marrow B-cells revealed recurrent KMT2D and CARD11 gene mutations in CAD patients [61]. The findings of a current study using cytogenetic microarrays and exome sequencing allowed for the identification of highly recurrent increases of chromosomes 3 and 12 or 18 in CAD-associated lymphoproliferative disease [62]. Furthermore, these genetic features of chromosome instability were similar to those demonstrated in nodal and extranodal marginal zone lymphoma (MZL) [63]. Genetic predisposition and immune dysregulation have also been reported in autoimmune phenomena in patients with chronic lymphocytic leukemia (CLL). It was observed that almost all patients with CAD presented with monoclonal antibodies encoded by the *IGHV4–34* gene responsible for the binding of I antigen [64–66]. Furthermore, microRNAs (miRNAs) are another factor implied in the gene expression disturbances found in CLL, and they have been found to be involved in both CLL and autoimmune cytopenia pathogenesis [64, 67, 68]. Regarding AIHA secondary to CLL, abnormalities in the regulatory mechanisms of the immune response were observed, including down-regulation of miRNAs [69], the presence of autoreactive polyclonal B cells (mainly IgG class) and neoplastic monoclonal B lymphocytes (mainly IgM class) [64], induction of autoreactive Th cells through B cell activator (BAFF) and a proliferation inducing ligand (APRIL), formation of nonfunctional Tregs [70], reduction of Toll-like receptors (TLR4), a lower expression of TLR2, and an increases of TLR7, TLR9, and TLR10 [71].

### AIHA characteristic

**Fig. 3:**
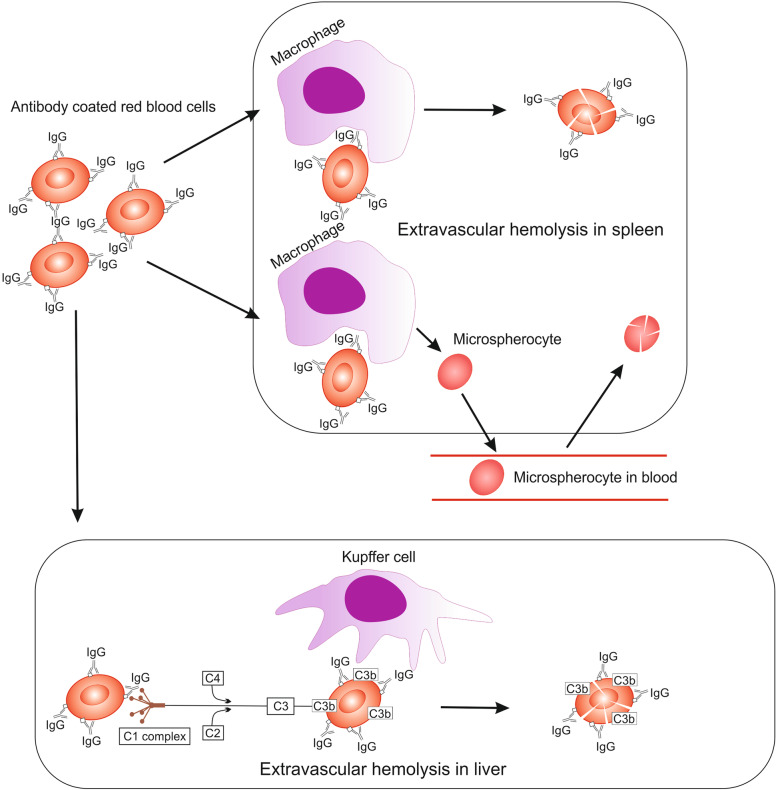
Hemolysis in the course of warm AIHA: the main pathological mechanisms and sites of hemolysis, adapted and modified from Berentsen and Sundic [72]. Antibody-coated RBCs are ingested and degraded by macrophages or destroyed by antibody dependent cell cytotoxicity in the spleen. Some of the erythrocytes are only partially phagocytosed and form microspherocytes which return to the circulation. Microspherocytes have rigid cellular membrane and lack flexibility which causes their sequestration in the spleen. Weak activation of complement by Ig G antibodies leads to extravascular hemolysis in the liver

The clinical picture of AIHA and the main pathological mechanisms differ slightly depending on the type of AIHA. In wAIHA (as with other types of AIHA), slight hemolysis may occur unnoticed, but increased hemolysis may lead to severe tissue hypoxia. Significant yellowing of the skin is observed with extensive hemolysis. Mild to moderate splenomegaly is often observed in active hemolysis, but disproportionate splenomegaly or nodular splenomegaly is characteristic in secondary forms, especially in lymphoproliferative diseases [9, 10, 32]. Autoantibodies in wAIHA, usually of class IgG, and/or complement, are attached to and destroy RBCs at about 37 °C, mainly in the process of extravascular hemolysis in the spleen. Additionally, IgG antibodies also have the ability to weakly activate complement and deposit C3 fragments on RBCs, which leads to their destruction by Kupffer cells in the liver (Fig. 3). Moreover, activation of the terminal complement pathway can lead to the formation of a membrane attack complex (C5b-9; MAC) on the surface of RBCs and cause intravascular hemolysis [9, 73]. Hence, during diagnostics, DAT is positive for IgG only or for IgG ± C3d, while cold agglutinins are negative (Table 2).

**Table 2:** Serologic characteristics of different types of AIHA [3, 11, 74]

Type of AIHA	Antibody type	Typical DAT	RBC eluate	Antigen specifity	Antibody titre at 4 °C
Warm AIHA	IgG (rarely IgA or IgM)	IgG or IgG + C3	IgG	panreactive	–
CAD	IgM	C3	nonreactive	usually anti-I^a^	usually > 1:500
PCH	biphasic IgG	C3	nonreactive	usually anti-P	< 1:64
Mixed AIHA	IgG, IgM	IgG + C3	IgG	usually lack specifity of warm IgG, cold antibody differently^b^	cold antibodies < 1:64
DIHA	Ig G	IgG or IgG + C3	IgG	often Rh-related	–

^a^sometimes anti-i, rarely anti-Pr

^b^anti I, anti-i or lack specificity

In CAD, symptoms are associated with temperature fluctuations, and rapid cooling can trigger hemolysis. Usually, symptoms associated with the presence of anemia dominate. Under the influence of cold, bruising and/or redness of the skin on distal parts of the body appear, i.e. acrocyanosis. Prolonged cold exposure may result in ischemia and necrosis [74].

**Fig. 4:**
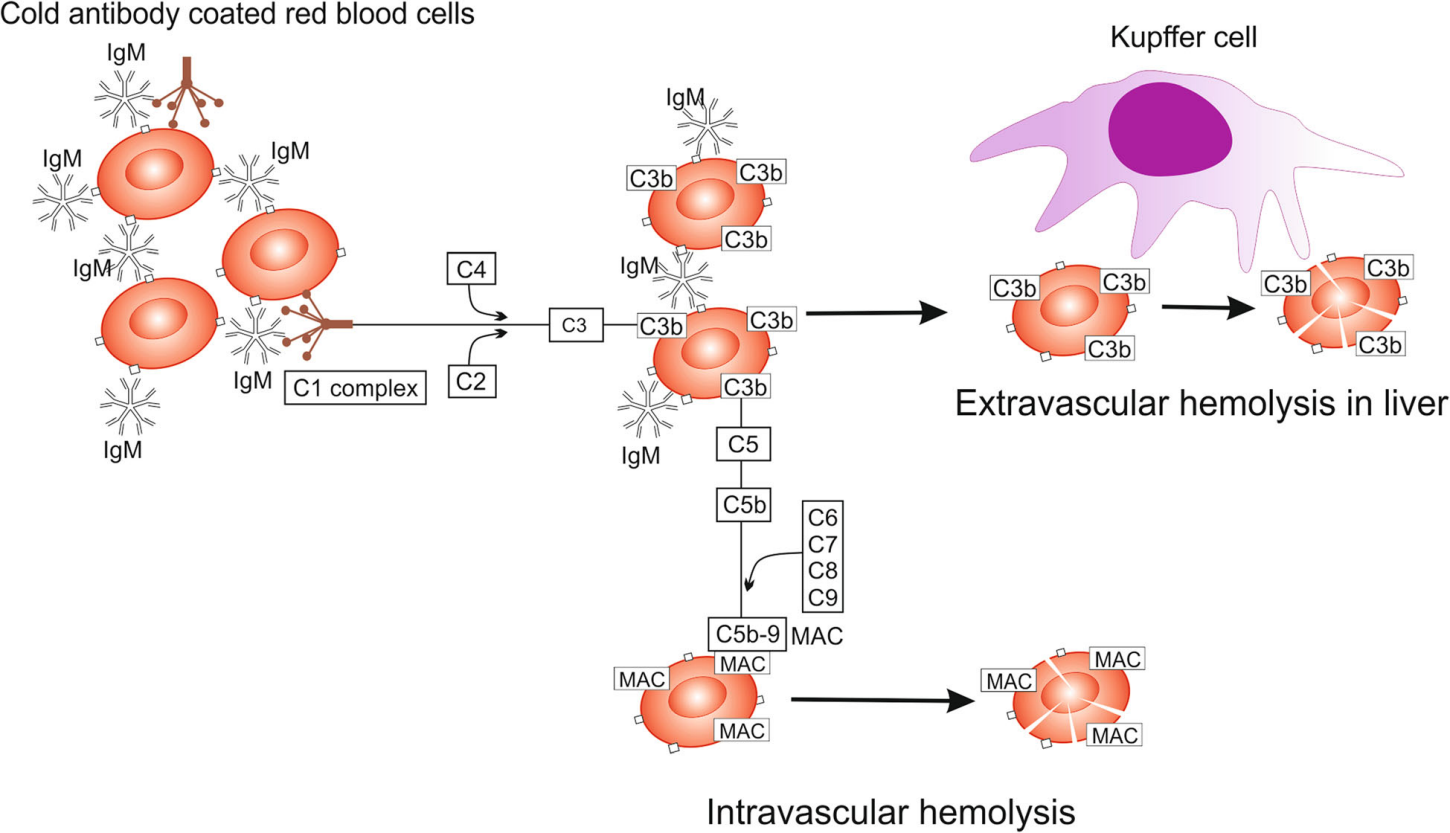
Hemolysis in CAD, adapted and modified from Berentsen et al. [72, 74]. Activation *of the* complement cascade caused by the reaction of cold agglutinins with RBCs leads to intravascular and extravascular hemolysis*.* Extravascular hemolysis is associated with complement activation and destruction of RBCs by the mononuclear phagocytic system, mainly in the liver. Intravascular hemolysis is a result of the formation of membrane attack complex (MAC), composed of C5b, C6, C7, C8, and C9

Cooling of distal parts of the body, such as fingers, nose, and ears, leads to the activation and binding of IgM autoantibodies to the erythrocyte membrane, followed by agglutination of RBCs (Fig. 4). The antigen-IgM complex present on the red blood cell binds the C1 component of the complement, which leads to the activation of the complement pathway and formation of the C3b component. When the blood temperature rises to about 37 °C IgM antibodies detach from the complex, but the C3b component remains on the erythrocyte membrane. Enzymatic conversion of the C3b component occurs on non-hemolyzed cells, and the C3d component is detected in DAT [75, 76].

The mixed form of AIHA accounts for less than 10% of all cases, with the course of disease usually chronic and not associated with cold. Antibody activation occurs in a wide temperature amplitude (usually > 30 °C) [73, 77].

PCH should be considered in the evaluation of any patient under 18 years old with hemolysis associated with exposure to cold and concomitant infections. In addition to the symptoms of the underlying disorder, the clinical manifestations of PCH include back or leg pain, abdominal cramping, fever or chills, jaundice, and dark (red to brown) urine especially at the beginning of urination [78, 79]. Since the hemolysis is mainly intravascular, patients with PCH do not always have severe signs or significant clinical symptoms. PCH is caused by polyclonal IgG type antibodies that bind to RBCs in cold temperatures (colder than normal body temperature) and fix complement causing complement-mediated intravascular hemolysis upon reheating. The thermal amplitude is most commonly < 20 °C [20, 73, 77].

### AIHA diagnosis

In AIHA, as in the course of other hemolytic anemias, normocytic anemia with spherocytes is found in the peripheral blood smear. Reticulocytosis (although reticulocytopenia sometimes occurs) is a typical finding in hemolytic anemia but not a specific marker and indicates an active and accelerated, compensatory production of erythrocytes in the bone marrow in response to hemolysis. The use of the bone marrow responsiveness index (BMRI) - calculated as the absolute reticulocyte count x patient’s Hb/normal Hb - in the assessment of insufficient erythropoiesis has been proposed [80]. Increased indirect (unconjugated) bilirubin levels, low or absent serum haptoglobin, elevated lactate dehydrogenase (LDH) and an increase in urinary urobilinogen are all hallmarks of hemolysis. Hemoglobinuria, which is an early symptom, indicates intravascular hemolysis. After about a week, hemosiderin can be detected in the urine [80]. The DAT confirms the immune mechanism. Sometimes low autoantibody titeres below the threshold of the test or no appropriate antibody testing can give a negative DAT despite the presence of AIHA. 5–10% of AIHA cases fail to obtain a DAT positive despite highly sensitive tests [2].

In the DAT, monospecific anti-globulins are used to detect autoantibodies directed against immunoglobulins, particularly IgG (IgG1 or IgG3) and a fragment of the third complement of complements C3d and C3c, or both, on the RBC surface. The study uses kits containing anti-IgG monospecific antibodies specific for anti-IgA, anti-IgM, anti-C3d, and anti-C3c. In contrast to the DAT, confirmation of free anti-RBC antibodies in the patient’s serum is possible by means of an indirect antiglobulin test (IAT; indirect Coombs test), whereas free IgM antibodies are examined in a low ionic strength solution (LISS) or through cold washing, which may overcome low-affinity autoantibodies. Obtaining the eluate by detaching antibodies from RBCs and assessing their reaction with donor erythrocytes confirms the presence of these autoantibodies. Meanwhile, cold agglutinin titer is a diagnostic test for CAD. Autoantibodies are monoclonal in CAD and in CAS secondary to lymphoma, but polyclonal in CAS secondary to infection [74]. A predominance of kappa chains (κ) appears in CAD monoclonal IgM, sometimes a few percent of lambda light chains (λ) are possible [22, 74]. For persons under the age of 18, with atypical serology and/or hemoglobinuria or cold-induced symptoms (acrocyanosis, livedo reticularis, or Raynaud phenomenon), a Donath-Landsteiner test is performed [9, 73, 77]. In the differential diagnosis, secondary causes of AIHA and underlying conditions and medications should be considered. Due to the significantly increased risk of venous thromboembolism in AIHA [81], immediate diagnosis is recommended. Bone marrow examination is indicated in CAD patients aged > 60 years, clinical presentation (weight loss, lymphadenopathy or hepatosplenomegaly) and/or peripheral blood smear abnormalities (lymphocytosis, and/or cytopenias) to exclude primary CAD-associated lymphoproliferative disorders [73]. Bone marrow evaluation is also recommended in relapsed or refractory wAIHA and in CAD at diagnosis [29]. Bone marrow studies in patients with primary AIHA have yielded interesting observations. Histopathological examination of bone marrow in 40 out of 54 patients with primary CAD revealed intraparenchymatous nodules with small uniform monoclonal B cells and sometimes scattered B-cells. The bone marrow image and genetic test were different from the known B-cell lymphoma [82]. In a study of 47 patients with different types of AIHA, bone marrow fibrosis (BMF) was found in more than 1/3 of the patients, while at least 2/3 of them had increased bone marrow cellularity (not only the erythroid lineage) and dyserytropoiesis [83]. Patients with fibrosis, hypercellularity and dyserytropoiesis more often required second and subsequent treatment lines [83]. In another study involving 99 patients with primary AIHA, a paroxysmal nocturnal hemoglobinuria (PNH) clone was found in 1/3 of them. Patients with accompanying PNH clones had higher LDH and a higher hemolytic pattern [84].

### AIHA treatment

AIHA treatment requires an individual approach to each patient, sometimes with repeated evaluation of the clinical condition and modification of therapy. Elderly patients have a lower tolerance for anaemia and therefore more often require treatment, and the treatment is more likely to have adverse drug reactions, drug interactions and therapy toxicity. Treatment depends on the type of AIHA, the presence and severity of clinical symptoms, the underlying diseases that caused the AIHA, and the presence of concomitant comorbidities. In general, symptomatic anemia is primarily an indication for therapy in both newly diagnosed and persistent AIHA. In the treatment of secondary AIHA, it is crucial to treat any underlying autoimmune diseases.

For all symptomatic cases, regardless of the severity of AIHA, each patient should receive folic acid and any other vitamin supplements necessary. Red blood cell transfusions should be limited only to critical cases with severe anemia (hemoglobin < 6 g/dL) and/or hemodynamically unstable patients, which is particularly common when treating the elderly [9]. Prevention of gastrointestinal bleeding, osteoporosis and *Pneumocystis jirovecii* infection should also be considered in patients on chronic GKS therapy [3]. Prophylaxis of VTE should be considered in inpatients and outpatients in the presence of hemolysis and additional risk factors for VTE. This is often considered during acute hemolysis or after a splenectomy [3, 81]. Intravenous immunoglobulin, plasma exchange, emergency splenectomy or partial splenic embolization are rescue therapies for emergency situations. In patients with severe AIHA, with a frequent need for blood transfusions and significant reticulcytopenia, an erythropoiesis-stimulating agent may be considered [3, 85].

Infections may underlie secondary AIHA, or may also be a result of therapy, and worsen the course of AIHA. Therefore, active testing for and prompt treatment and prevention of infections (especially Mycoplasma pneumoniae, hepatitis B and C, HIV, EBV, CMV, parvovirus B19, tuberculosis) is recommended [3, 29, 74, 86]. B-cell depleting therapies (e.g. RTX) are associated with possible reactivation of a hepatitis B infection, therefore antiviral prophylaxis is advised [87]. Before splenectomy, vaccinations against *Haemophilus influenzae*, Meningococcus and Pneumoccoccus species are recommended [9].

### Warm AIHA treatment

GKS is a first-line therapy in newly diagnosed patients with wAIHA [3]. Prednisone is usually administered orally at a starting dose of 1 mg/kg, or optionally an equivalent dose of methylprednisolone administered intravenously. Approximately 80% of patients improve within 2–3 weeks [88, 89]. Long-term remissions after GKS withdrawal are achieved only in 20–30% of cases [30, 32]. When there is no improvement after about 3 weeks of GKS therapy, other medications are usually added. It has been reported that combined therapy of rituximab (RTX - a chimeric human IgG1-κ monoclonal antibody against the protein CD20) with GKS, as a first-line treatment, gives a better response than GKS monotherapy [90, 91]. Furthermore, based on a recently published meta-analysis, RTX treatment is more effective than treatments without RTX, for AIHA and microangiopathic hemolytic anemia [92].

It is important to note that adding rituximab therapy reduces the need for repeated GKS treatment, also in steroid-dependent patients, which is particularly beneficial for old patients with co-morbidities. In these groups especially, GKS therapy increases the risk of numerous adverse effects when given for a prolonged period, including gastrointestinal bleeding, osteoporosis, diabetes, etc. In order to reduce the effects of chronic steroid therapy, it is recommended to prevent osteoporosis in all patients over 50 years of age and proton pump inhibitors in patients aged over 60 years old and regardless of the age when using non-steroidal anti-inflammatory drugs (NSAID), anticoagulant or antiplatelet agents [77]. RTX gives a response rate of 70–80% in the second-line treatment of AIHA [91, 93, 94] administered usually at doses of 375 mg/m^2^ once weekly for 4 weeks. A 10-year prospective study showed the efficacy (both short-term and long-term outcome) of low doses of RTX (100 mg fixed dose once weekly for 4 weeks) in primary AIHA, with better effects in wAIHA [95], however, with a significant rate of relapses within 2 years. The effectiveness and safety of RTX therapy has also been demonstrated in older patients [96]. Based on a study of 23 elderly patients (median age 78 years) with refractory wAIHA, the response was 86.9% and median overall survival (OS) was 87 months [96]. Unfortunately, a significant percentage had relapses within 2 years. An extremely rare complication of RTX therapy is progressive multifocal leukoencephalopathy caused by JV virus reactivation [9].

Splenectomy may be considered as the third line option of wAIHA treatment [3]. Of patients with primary wAIHA, 40–90% achieve a response after splenectomy, but relapse occurs in about 80% of patients [32, 80, 97, 98]. Splenectomy is an invasive and irreversible form of treatment and carries an increased risk of thrombosis and encapsulated bacterial infections. In recent years, however, the risk of complications and mortality associated with splenectomies has been reduced, however, there are no data on the efficacy and safety of this therapy in elderly wAIHA patients [99].

Immunosuppressive drugs, especially azathioprine and mycophenolate-mofetil are an alternative to RTX in wAIHA associated with SLE [3]. Furthermore, previously suggested treatment lines such as cyclophosphamide or autologous bone marrow transplantation [9] have a weaker evidence base and carry greater risks of potential complications [3]. Combination therapy with various drugs shows better results, as demonstrated by Kaufman and colleagues in the case of immune cytopenias in CLL. CTX plus rituximab plus dexamethasone gave a 100% response [100]. Treatment of CLL associated AIHA depends on the stage of CLL. Management of AIHA in the early stage of CLL is the same as in primary AIHA. Patients with CLL requiring therapy (stage III/IV according to Rai or Binnet C stage) or who do not respond to GKS and RTX need CLL target therapy [3].

### CAD and PCH treatment

Treatment of mild CAD is not recommended, especially for patients when Hb is > 10 g/dl. Patients with coexisting diseases such as ischaemic heart disease, cardiomyopathy, or chronic obstructive pulmonary disease and symptoms indicating hypoxia may require treatment with higher levels of Hb [3]. In CAD patients, thermal protection of distal parts of the body against cooling is advised. Rituximab alone or in combination with other drugs, especially bendamustine, is currently the first-line treatment for severe CAD [3, 74]. Based on two prospective RTX studies, an overall response rate (ORR) of between 45 and 54% was achieved, but remissions were not durable [86, 101]. Berentsen reported the longest median response of 11 months [22]. Based on a meta-analysis by Reynaud et al. the ORR for RTX in CAD was 57%, but the complete response rate (CRR) was only 21% [94]. Combined therapy of RTX with bendamustine gave a response of 71%, including 40% CRR with acceptable toxicity in fit patients [102]. In contrast, after immunochemotherapy based on RTX with fludarabine the observed response rate was 76% of patients (including 21% CRR) and with a median remission of 66 months [103]. Another treatment option is eculizumab, a humanized monoclonal antibody that inhibits C5 level complement. In a prospective, non-randomized phase 2 trial with eculizumab in patients with CAD, a decrease in hemolysis severity and a decreased necessity for blood transfusion were demonstrated [104]. The high efficacy of eculizumab in fulminant hemolytic anemia in primary CAD has also been reported [105]. There have also been reports of the effective inhibition of hemolysis by eculizumab in severe, idiopathic wAIHA cases which did not respond to GKS, intravenous immunoglobulin, mycophenolate mofetil and RTX [106]. In the case of severe hemolysis, plasmapheresis is considered, which is highly effective in removing cold agglutinins, however, only for a short time. In order to avoid complement activation induced by plasma infusion, plasmapheresis must be performed with albumin as a replacement fluid [107].

Splenectomy is not recommended for CAD because the liver is the main location for extravascular hemolysis. Transfusion of RBCs using warmer blood may be necessary when indicated in severe symptomatic anemia [74].

### Future prospects of AIHA therapy

**Fig. 5:**
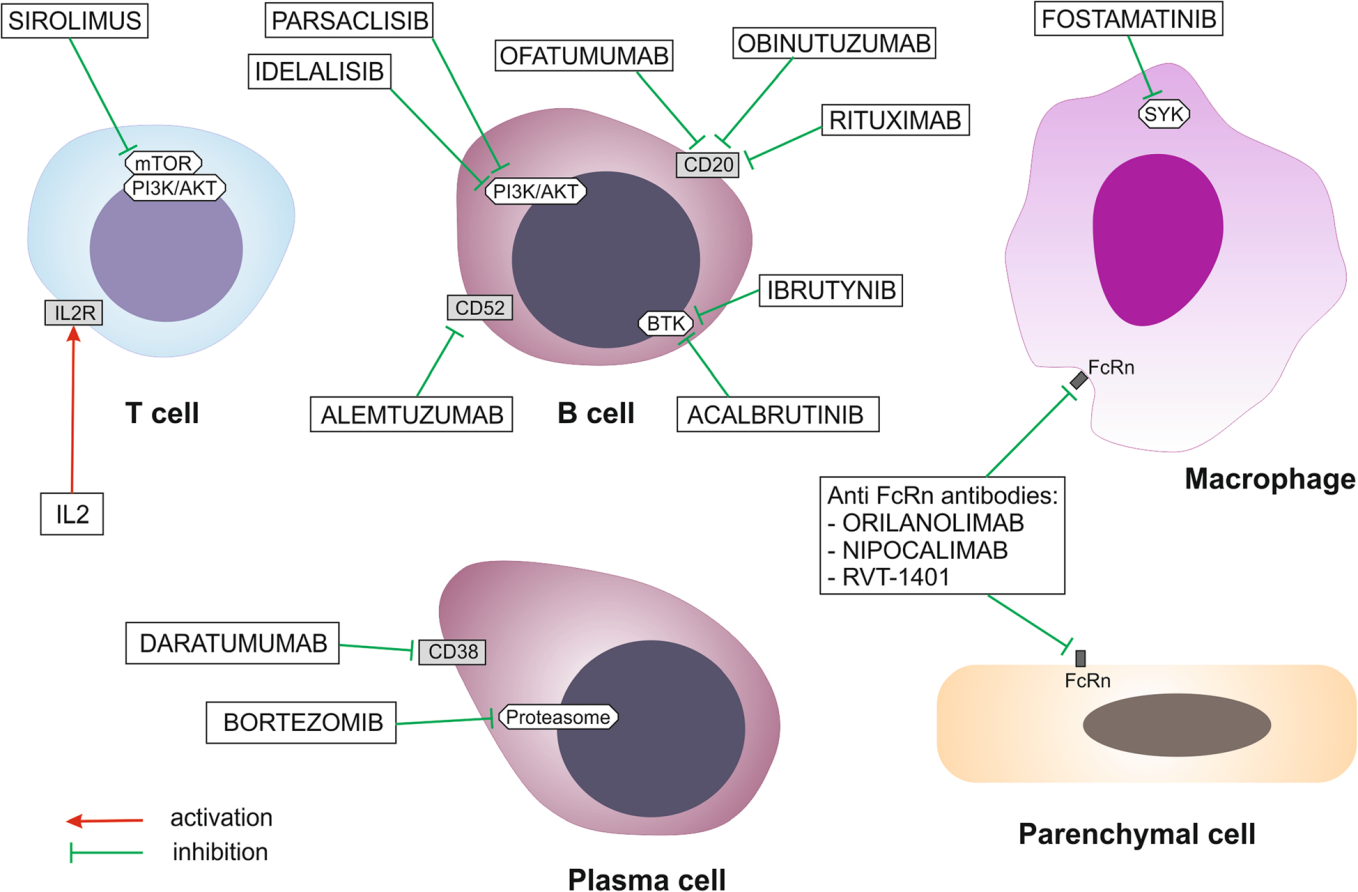
New, potential drugs targeting mainly immune cells, adapted and modified from Michalak [108]. BTK - Bruton’s tyrosine kinase, CD - cluster of differentiation - cell surface antigen, FcRn - neonatal crystallizable fragment of the receptor, IL 2 - interleukin 2, IL 2R - interleukin 2 receptor, mTOR - mammalian target of rapamycin kinase, PI3K - hosphatidylinositol 3-kinase, PI3K/AKT - intracellular signaling pathway, SYK - spleen tyrosine kinase, SYNT001 - monoclonal antibody (Orilanolimab)

Along with a better understanding of AIHA pathogenesis, a need for new and more effective AIHA therapies arises. The targets of new therapies in AIHA are mainly the B lymphocytes, T lymphocytes and the complement cascade, but they also include the spleen tyrosine kinase (SYK) on macrophages and the neonatal crystallizable fragment of the receptor (FcRn) present on many cells, including endothelial cells and macrophages (Fig. 5). New groups of drugs are in different phases of clinical trials, as shown in Table 3.

**Table 3:** Ongoing clinical trials of new drug groups in AIHA

Intervention/ treatment	Group of agents	Condition or disease	Phase of study	**ClinicalTrials.gov**
Sirolimus plus ATRA	mTOR inhibitor plus tretinoin	refractory AIHA	2 and 3	NCT04324411
Idelalisib vs. ibrutinib	PI3Kδ inhibitor vs. BTK inhibitor	autoimmune cytopenia in the course of CLL	retrospective	NCT03469895
Parsaclisib	PI3Kδ inhibitor	AIHA	2	NCT03538041
Ibrutinib	BTK inhibitor	steroid refractory warm AIHA	2	NCT03827603
		refractory/relapsed AIHA	2	NCT04398459
Ibrutinib or idelalisib	BTK inhibitor	AIHA associated with CLL	retrospective	NCT03469895
Interleukine-2	cytokine	resistant, warm AIHA	1 and 2	NCT02389231
Low dose rituximab plus alemtuzumab	anti CD20 antibody plus anti CD52 antibody	refractory autoimmune cytopenias	2 and 3	NCT00749112
Fostamatinib	SYK inhibitor	warm AIHA	3	NCT03764618
			2	NCT02612558
SYNT001 (ALXN1830)	anti-FcRn antibody	warm AIHA	1 and 2	NCT03075878
SYNT001 (ALXN1830) vs. placebo			2	NCT04256148
M281			2 and 3	NCT04119050
RVT-1401			2	NCT04253236
Levamisole plus prednisolone	immunomodulatory drug plus GKS	warm AIHA	2	NCT01579110
BIVV009 (Sutimlimab)	complement C1 inhibitor	CAD	3	NCT03347422
			3	NCT03347396
APL2	complement C3 inhibitor	warm AIHA, CAD	2	NCT03226678

*AIHA* autoimmune hemolytic anemia, *ATRA* all trans-retinoic acid, *BTK* Bruton’s tyrosine kinase, *CLL* chronic lymphocytic leukemia, *FcRn* neonatal crystallizable fragment of the receptor, *mTOR* mammalian target of rapamycin kinase, *PI3K* phosphatidylinositol 3-kinase, *SYK* spleen tyrosine kinase

Regarding treatment modulating the function of lymphocytes, the interleukin 2 receptor (IL2R), the phosphatidylinositol 3-kinase (PI3K), and mTOR (mammalian target of rapamycin) axis are considered to be promising treatment targets. Sirolimus and IL2 disrupt the function of pathological lymphocytes T. Idelalisib and PI3K δ inhibitor alter the PI3K axis and lead to pathological lymphocyte B dysregulation. Sirolimus has been shown to be effective in the management of AIHA in children after transplantation [109] and resistant AIHA [110]. Plasma cells are especially targeted by proteasome inhibitors and anti-CD38 antibodies. In a retrospective study, bortezomib (the first registered proteasome inhibitor) together with other drugs elicited a positive response in patients with refractory wAIHA [111, 112]. Daratumumab (anti CD38 monoclonal antibody) has shown a rapid and sustained response in the treatment of refractory AIHA after HSCT in children [113] and in adults [114]. Proliferation disorder and apoptosis of pathological B lymphocytes is obtained by means of bruton tyrosine kinase (BTKs) inhibitors and antibodies directed against CD20 or CD52. Recent single reports indicate that acalabrutinib, a new BTK inhibitor, used in therapy for relapsed/refractory CLL, may also reduce the incidence of concomitant autoimmune cytopenia including AIHA [115]. New anti-CD20 antibodies with potential significance for AIHA have been developed, including ofatumumab [116]. It has been shown that treatment based on alemtuzumab (anti CD52 monoclonal antibody) and RTX induced a short-term response in a group of 8 patients with primary AIHA [117].

Macrophages are the target of SYK inhibitors and fostamatinib is the first drug from this group. Fostamatinib impairs macrophage function and blocks off the phagocytosis of RBCs coated with antibodies. Orilanolimab (SYNT001) is a humanized monoclonal antibody which blocks the interaction between the neonatal crystallizable fragment receptor (FcRn) and IgG. FcRn is responsible for salvaging IgG from lysosomal degradation. By preventing FcRn/IgG binding, orilanolimab blocks the FcRn-mediated rescue of IgG, enables IgG degradation and also reduces the serum concentrations of the total IgG [118]. In a review of current clinical trials, there are other anti-FcRn monoclonal antibodies that reduce the amount of IgG: M281 (nipocalimab) and RVT-1401.

Complement modulation in AIHA therapy is currently possible at 3 levels: at the C1 complex level (which includes C1q, C1 r, C1 s), at the C3 and at the C5 convertases of the complement pathway (Table 4).

**Table 4:** Complement modulation in AIHA treatment: now and in the future

Level of complement pathway modulation	Novel agents	Comments
C1 complex (C1q, C1r, C1s)	ANX005	anti-C1q monoclonal antibody
	TNT003	anti-C1s monoclonal antibody
	PIC1	peptide inhibitor
	Sutimlimab	anti-C1s monoclonal antibody
C3 complement	Compstatin Cp40	long-acting form polyethylene glycol
	Pegcetacoplan (APL2)	pegylated compstatin analog
C5 complement	Eculizumab	anti-C5 monoclonal antibody

Drugs which block the initial classical complement pathway, but also the lectin pathways, include peptide C1 complement inhibitors (PIC1) [119]. TNT003 is a murine monoclonal antibody which targets the specific serine protease C1s, and its effectiveness has been investigated *in vitro* and *in vivo* [120, 121]. Sutimlimab - a humanized anti-C1 monoclonal antibody (formerly called BIVV009 or TNT009) rapidly inhibits hemolysis in patients with CAD, increases Hb and resolves anemia, furthermore the patients remain transfusion-free [122, 123].

No clinical trial results have yet been published with regard to ANX005 (a humanized anti-C1q antibody) use in CAD. For now the studies have been conducted *in vitro* with mouse erythrocytes and also with human serum patients with CAD [124]. Inhibiton of C3 activation of the complement system does not prevent hemolysis via the classical pathway, but it can eliminate hemolysis mediated by an alternative pathway. The effects of Compstatin Cp40, which inhibits opsonization by C3b of erythrocytes, have been shown in *in vitro* and preclinical studies [125–127]. Eculizumab blocks the complement at the C5 level and is sometimes used in persistent, severe AIHA [104–106]. In silico and *in vitro* research is ongoing to find new compounds that could inhibit the initial complement pathways. Studies showed that potentially the highest activity compound was 1,2,4-triazole [128].

## Conclusions

AIHA is a rare, heterogeneous group of diseases in which, despite the wealth of research on its pathogenesis and treatment, there are still many issues to be resolved. The complex diagnostics and treatment of AIHA require an individual approach. In refractory or recurrent cases, sequential treatment lines are used. AIHA is still a disease that causes difficulties in the treatment and this is why it is important to develop research into new drugs in AIHA. Research into new drugs in the treatment of AIHA targeting B cell and plasma cells, T cells and macrophages is developing intensively. An increase in knowledge on the role of the complement in autohemolysis has contributed to the development of drugs that inhibit the complement pathways at various levels. It would be advisable to keep an AIHA registry in each country, which would facilitate data collection, monitoring and awareness of this disease. To collect data, it would be necessary to use not only consistent terminology, including recognition and classification criteria, but also uniform response criteria. Studies comparing the effects of different therapies on the quality of life and the need for blood transfusions would be useful. Further clinical and basic research on treatment options is warranted and the paucity of data on special characteristics of older patients requires more attention.

## Data Availability

Not applicable.

## Abbreviations

AIHA: Autoimmune hemolytic anemia
ALPS: Autoimmune lymphoproliferative syndrome
ADCC: Antibody dependent cel cytotoxity
APL 2: Complement C3 inhibitor
ATRA: All trans-retinoic acid
BIVV009: Complement C1s inhibitor
BTK: Bruton’s tyrosine kinase
CAD: Cold agglutinin disease
CD: Cluster of differentiation - cell surface antigen
CAS: Cold agglutinin syndrome
CLL: Chronic lymphocytic leukemia
CVID: Common variable immunodeficiency
CRR: Complete response rate
Compstatin Cp40: Complement C3 inhibitor
DAT: Direct antiglobulin test
DIIHA: Drug-induced immune hemolytic anaemia
FcRn: Neonatal crystallizable fragment of the receptor
GKS: Glicocorticosteroid
HAART: Highly active antiretroviral therapy
HIV: Human immunodeficiency virus
HSCT: Haematopoietic stem cell transplantation
IL2: Interleukin 2
IL2R: Interleukin 2 receptor
ICIs: Immune checkpoint inhibitors
MAC: Membrane attack complex
mTOR: Mammalian target of rapamycin kinase
NHL: Non-Hodgkin lymphoma
OSR: Overall survival rate
PCH: Paroxysmal cold hemoglobinuria
PBC: Primary biliary cirrhosis
PIC1: Peptide inhibitor of complement C1
PI3K: Phosphatidylinositol 3-kinase
PI3K/AKT: Intracellular signaling pathway
RBCs: Red blood cells
RTX: Rituximab
SLE: Systemic lupus erythematosus
SYK: Spleen tyrosine kinase
SYNT001: Monoclonal antibody
TNT003: Anti-C1s antibody
Tfh: T follicular helper cells
Tfr: T follicular regulatory cells
Tregs: Regulatory T cells
VTE: Venous Thromboembolism
wAIHA: Warm AIHA
HBV: Hepatitis B virus

## References

[1] Goronzy JJ, Li G, Yang Z, Weyand CM (2013). The janus head of T cell aging - autoimmunity and immunodeficiency. Front Immunol.

[2] Barcellini W, Fattizzo B, Zaninoni A (2018). Current and emerging treatment options for autoimmune hemolytic anemia. Expert Rev Clin Immunol.

[3] Jäger U, Barcellini W, Broome CM, Gertz MA, Hill A, Hill QA (2020). Diagnosis and treatment of autoimmune hemolytic anemia in adults: Recommendations from the First International Consensus Meeting. Blood Rev.

[4] Hill QA, Hill A, Berentsen S (2019). Defining autoimmune hemolytic anemia: a systematic review of the terminology used for diagnosis and treatment. Blood Adv.

[5] Lazarian G, Quinquenel A, Bellal M, Siavellis J, Jacquy C, Re D (2020). Autoimmune haemolytic anaemia associated with COVID-19 infection. Br J Haematol.

[6] Garratty G (2012). Immune hemolytic anemia caused by drugs. Expert Opin Drug Saf.

[7] Hill QA, Stamps R, Massey E, Grainger JD, Provan D, Hill A (2017). Guidelines on the management of drug-induced immune and secondary autoimmune, haemolytic anaemia. Br J Haematol.

[8] Yen Y-F, Lan Y-C, Huang C-T, Jen I-A, Chen M, Lee C-Y (2017). Human immunodeficiency virus infection increases the risk of incident autoimmune hemolytic Anemia: a population-based cohort study in Taiwan. J Infect Dis.

[9] Brodsky RA (2019). Warm Autoimmune Hemolytic Anemia. N Engl J Med.

[10] Swiecicki PL, Hegerova LT, Gertz MA (2013). Cold agglutinin disease. Blood..

[11] Garratty G (2010). Immune hemolytic anemia associated with drug therapy. Blood Rev.

[12] Podjasek JC, Abraham RS (2012). Autoimmune cytopenias in common variable immunodeficiency. Front Immunol.

[13] Bride K, Teachey D (2017). Autoimmune lymphoproliferative syndrome: more than a FAScinating disease. F1000Res.

[14] Michniacki TF, Ebens CL, Choi SW (2019). Immune-mediated Cytopenias after hematopoietic cell transplantation: pathophysiology, clinical manifestations, diagnosis, and treatment strategies. Curr Oncol Rep.

[15] Kruizinga MD, van Tol MJD, Bekker V, Netelenbos T, Smiers FJ, Bresters D (2018). Risk factors, treatment, and immune Dysregulation in autoimmune Cytopenia after allogeneic hematopoietic stem cell transplantation in pediatric patients. Biol Blood Marrow Transplant.

[16] Lv W, Qu H, Wu M, Fan Z, Huang F, Xu N (2019). Autoimmune hemolytic anemia after allogeneic hematopoietic stem cell transplantation in adults: a southern China multicenter experience. Cancer Med.

[17] Barcellini W, Fattizzo B, Zaninoni A (2019). Management of refractory autoimmune hemolytic anemia after allogeneic hematopoietic stem cell transplantation: current perspectives. J Blood Med.

[18] Hansen DL, Möller S, Andersen K, Gaist D, Frederiksen H (2020). Increasing incidence and prevalence of acquired hemolytic Anemias in Denmark, 1980-2016. Clin Epidemiol.

[19] Packman CH (2008). Hemolytic anemia due to warm autoantibodies. Blood Rev.

[20] Berentsen S, Röth A, Randen U, Jilma B, Tjønnfjord GE (2019). Cold agglutinin disease: current challenges and future prospects. J Blood Med.

[21] Sokol RJ, Hewitt S, Stamps BK (1981). Autoimmune haemolysis: an 18-year study of 865 cases referred to a regional transfusion Centre. Br Med J (Clin Res Ed).

[22] Berentsen S, Ulvestad E, Langholm R, Beiske K, Hjorth-Hansen H, Ghanima W (2006). Primary chronic cold agglutinin disease: a population based clinical study of 86 patients. Haematologica..

[23] Göttsche B, Salama A, Mueller-Eckhardt C (1990). Donath-Landsteiner autoimmune hemolytic anemia in children. A study of 22 cases. Vox Sang.

[24] Leibrandt R, Angelino K, Vizel-Schwartz M, Shapira I (2018). Paroxysmal Cold Hemoglobinuria in an Adult with Respiratory Syncytial Virus. Case Rep Hematol.

[25] Fülöp T, Larbi A, Pawelec G (2013). Human T cell aging and the impact of persistent viral infections. Front Immunol.

[26] Wada T, Koyama D, Kikuchi J, Honda H, Furukawa Y (2015). Overexpression of the shortest isoform of histone demethylase LSD1 primes hematopoietic stem cells for malignant transformation. Blood..

[27] Gusev GP, Govekar R, Gadewal N, Agalakova NI (2017). Understanding quasi-apoptosis of the most numerous enucleated components of blood needs detailed molecular autopsy. Ageing Res Rev.

[28] Lupescu A, Bissinger R, Goebel T, Salker MS, Alzoubi K, Liu G (2015). Enhanced suicidal erythrocyte death contributing to anemia in the elderly. Cell Physiol Biochem.

[29] Barcellini W, Giannotta J, Fattizzo B. Autoimmune hemolytic anemia in adults: primary risk factors and diagnostic procedures. Expert Rev Hematol. 2020;13(6):585–97. 10.1080/17474086.2020.1754791 32274943

[30] Roumier M, Loustau V, Guillaud C, Languille L, Mahevas M, Khellaf M (2014). Characteristics and outcome of warm autoimmune hemolytic anemia in adults: new insights based on a single-center experience with 60 patients. Am J Hematol.

[31] Barcellini W, Zaninoni A, Fattizzo B, Giannotta JA, Lunghi M, Ferrari A (2018). Predictors of refractoriness to therapy and healthcare resource utilization in 378 patients with primary autoimmune hemolytic anemia from eight Italian reference centers. Am J Hematol.

[32] Barcellini W, Fattizzo B, Zaninoni A, Radice T, Nichele I, Di Bona E (2014). Clinical heterogeneity and predictors of outcome in primary autoimmune hemolytic anemia: a GIMEMA study of 308 patients. Blood..

[33] Russell PJ, Cunningham J, Dunkley M, Wilkinson NM (1981). The role of suppressor T cells in the expression of autoimmune haemolytic anaemia in NZB mice. Clin Exp Immunol.

[34] Iuchi Y, Kibe N, Tsunoda S, Suzuki S, Mikami T, Okada F (2010). Implication of oxidative stress as a cause of autoimmune hemolytic anemia in NZB mice. Free Radic Biol Med.

[35] Mqadmi A, Zheng X, Yazdanbakhsh K (2005). CD4+CD25+ regulatory T cells control induction of autoimmune hemolytic anemia. Blood..

[36] Richards AL, Kapp LM, Wang X, Howie HL, Hudson KE (2016). Regulatory T cells are dispensable for tolerance to RBC antigens. Front Immunol.

[37] Howie HL, Hudson KE (2018). Murine models of autoimmune hemolytic anemia. Curr Opin Hematol.

[38] Chatterjee S, Bhardwaj N, Saxena RK (2016). Identification of stages of Erythroid differentiation in bone marrow and erythrocyte subpopulations in blood circulation that are preferentially lost in autoimmune hemolytic Anemia in mouse. PLoS One.

[39] Chatterjee S, Saxena RK (2017). Binding of autoantibodies and apoptotic response in erythroid cells in the mouse model of autoimmune hemolytic anemia.

[40] Xu L, Zhang T, Liu Z, Li Q, Xu Z, Ren T (2012). Critical role of Th17 cells in development of autoimmune hemolytic anemia. Exp Hematol.

[41] Yao X, Li C, Yang J, Wang G, Li C, Xia Y (2016). Differences in frequency and regulation of T follicular helper cells between newly diagnosed and chronic pediatric immune thrombocytopenia. Blood Cells Mol Dis.

[42] Burenbatu, Borjigin M, Eerdunduleng, Huo W, Gong C, Hasengaowa (2015). Profiling of miRNA expression in immune thrombocytopenia patients before and after Qishunbaolier (QSBLE) treatment. Biomed Pharmacother.

[43] Zhang H-W, Zhou P, Wang K-Z, Liu J-B, Huang Y-S, Tu Y-T (2016). Platelet proteomics in diagnostic differentiation of primary immune thrombocytopenia using SELDI-TOF-MS. Clin Chim Acta.

[44] Gao Y, Jin H, Nan D, Yu W, Zhang J, Yang Y (2019). The role of T follicular helper cells and T follicular regulatory cells in the pathogenesis of autoimmune hemolytic Anemia. Sci Rep.

[45] Smirnova SJ, Sidorova JV, Tsvetaeva NV, Nikulina OF, Biderman BV, Nikulina EE (2016). Expansion of CD8+ cells in autoimmune hemolytic anemia. Autoimmunity..

[46] Tsiakalos A, Routsias JG, Kordossis T, Moutsopoulos HM, Tzioufas AG, Sipsas NV (2011). Fine epitope specificity of anti-erythropoietin antibodies reveals molecular mimicry with HIV-1 p17 protein: a pathogenetic mechanism for HIV-1-related anemia. J Infect Dis.

[47] Martinez V, Diemert M-C, Braibant M, Potard V, Charuel J-L, Barin F (2009). Anticardiolipin antibodies in HIV infection are independently associated with antibodies to the membrane proximal external region of gp41 and with cell-associated HIV DNA and immune activation. Clin Infect Dis.

[48] Barcellini W, Fattizzo B. The Changing Landscape of Autoimmune Hemolytic Anemia. Front Immunol. 2020;11:946. Available from: https://www.ncbi.nlm.nih.gov/pmc/articles/PMC7325906/. 10.3389/fimmu.2020.00946 PMC7325906 32655543

[49] Fattizzo B, Barcellini W (2019). Autoimmune Cytopenias in chronic lymphocytic leukemia: focus on molecular aspects. Front Oncol.

[50] Lisco A, Wong C-S, Price S, Ye P, Niemela J, Anderson M (2019). Paradoxical CD4 Lymphopenia in autoimmune Lymphoproliferative syndrome (ALPS). Front Immunol.

[51] Sánchez-Ramón S, Radigan L, Yu JE, Bard S, Cunningham-Rundles C (2008). Memory B cells in common variable immunodeficiency: clinical associations and sex differences. Clin Immunol.

[52] Iuchi Y, Okada F, Onuma K, Onoda T, Asao H, Kobayashi M (2007). Elevated oxidative stress in erythrocytes due to a SOD1 deficiency causes anaemia and triggers autoantibody production. Biochem J.

[53] Johnson RM, Goyette G, Ravindranath Y, Ho Y-S (2005). Hemoglobin autoxidation and regulation of endogenous H2O2 levels in erythrocytes. Free Radic Biol Med.

[54] Otsuki N, Konno T, Kurahashi T, Suzuki S, Lee J, Okada F (2016). The SOD1 transgene expressed in erythroid cells alleviates fatal phenotype in congenic NZB/NZW-F1 mice. Free Radic Res.

[55] Zhong H, Yazdanbakhsh K (2018). Hemolysis and immune regulation. Curr Opin Hematol.

[56] Buerck JP, Burke DK, Schmidtke DW, Snyder TA, Papavassiliou D, O’Rear EA (2019). A Flow Induced Autoimmune Response and Accelerated Senescence of Red Blood Cells in Cardiovascular Devices. Sci Rep.

[57] Leal JKF, Adjobo-Hermans MJW, Bosman GJCGM. Red Blood Cell Homeostasis: Mechanisms and Effects of Microvesicle Generation in Health and Disease. Front Physiol. 2018;9:703. Available from: https://www.ncbi.nlm.nih.gov/pmc/articles/PMC6002509/. 10.3389/fphys.2018.00703 PMC6002509 29937736

[58] Kriebardis AG, Antonelou MH, Stamoulis KE, Economou-Petersen E, Margaritis LH, Papassideri IS (2008). RBC-derived vesicles during storage: ultrastructure, protein composition, oxidation, and signaling components. Transfusion..

[59] Gao Y, Jin H, Tan H, Wang Y, Wu J, Wang Y, et al. The role of extracellular vesicles from stored RBC units in B lymphocyte survival and plasma cell differentiation. J Leukoc Biol. Available from: https://jlb.onlinelibrary.wiley.com/doi/abs/10.1002/JLB.1A0220-666R. 10.1002/JLB.1A0220-666R 32421907

[60] Michaux L, Dierlamm J, Wlodarska I, Stul M, Bosly A, Delannoy A (1995). Trisomy 3 is a consistent chromosome change in malignant lymphoproliferative disorders preceded by cold agglutinin disease. Br J Haematol.

[61] Małecka A, Trøen G, Tierens A, Østlie I, Małecki J, Randen U (2018). Frequent somatic mutations of KMT2D (MLL2) and CARD11 genes in primary cold agglutinin disease. Br J Haematol.

[62] Małecka A, Delabie J, Østlie I, Tierens A, Randen U, Berentsen S (2020). Cold agglutinin–associated B-cell lymphoproliferative disease shows highly recurrent gains of chromosome 3 and 12 or 18. Blood Adv.

[63] Bertoni F, Rossi D, Zucca E (2018). Recent advances in understanding the biology of marginal zone lymphoma. F1000Res.

[64] Potter KN, Hobby P, Klijn S, Stevenson FK, Sutton BJ (2002). Evidence for involvement of a hydrophobic patch in framework region 1 of human V4-34-encoded Igs in recognition of the red blood cell I antigen. J Immunol.

[65] Hu Y, Wang X, Yu S, Hou Y, Ma D, Hou M (2015). Neutralizations of IL-17A and IL-21 regulate regulatory T cell/T-helper 17 imbalance via T-helper 17-associated signaling pathway in immune thrombocytopenia. Expert Opin Ther Targets.

[66] Yu PB, Deng DY, Lai CS, Hong CC, Cuny GD, Bouxsein ML (2008). BMP type I receptor inhibition reduces heterotopic ossification. Nat Med.

[67] Strati P, Caligaris-Cappio F (2011). A matter of debate in chronic lymphocytic leukemia: is the occurrence of autoimmune disorders an indicator of chronic lymphocytic leukemia therapy?. Curr Opin Oncol.

[68] Grandjenette C, Kennel A, Faure GC, Béné MC, Feugier P (2007). Expression of functional toll-like receptors by B-chronic lymphocytic leukemia cells. Haematologica..

[69] Ferrer G, Navarro A, Hodgson K, Aymerich M, Pereira A, Baumann T (2013). MicroRNA expression in chronic lymphocytic leukemia developing autoimmune hemolytic anemia. Leuk Lymphoma.

[70] Muzio M, Bertilaccio MTS, Simonetti G, Frenquelli M, Caligaris-Cappio F (2009). The role of toll-like receptors in chronic B-cell malignancies. Leuk Lymphoma.

[71] Barcellini W, Imperiali FG, Zaninoni A, Reda G, Consonni D, Fattizzo B (2014). Toll-like receptor 4 and 9 expression in B-chronic lymphocytic leukemia: relationship with infections, autoimmunity and disease progression. Leuk Lymphoma.

[72] Berentsen S, Sundic T (2015). Red blood cell destruction in autoimmune hemolytic anemia: role of complement and potential new targets for therapy. Biomed Res Int.

[73] Hill A, Hill QA (2018). Autoimmune hemolytic anemia. Hematology Am Soc Hematol Educ Program.

[74] Berentsen S (2020). New insights in the pathogenesis and therapy of cold agglutinin-mediated autoimmune hemolytic Anemia. Front Immunol.

[75] Berentsen S (2014). Complement, cold agglutinins, and therapy. Blood..

[76] McNicholl FP (2000). Clinical syndromes associated with cold agglutinins. Transfus Sci.

[77] Hill QA, Stamps R, Massey E, Grainger JD, Provan D, Hill A (2017). The diagnosis and management of primary autoimmune haemolytic anaemia. Br J Haematol.

[78] Shanbhag S, Spivak J (2015). Paroxysmal cold hemoglobinuria. Hematol Oncol Clin North Am.

[79] Slemp SN, Davisson SM, Slayten J, Cipkala DA, Waxman DA (2014). Two case studies and a review of paroxysmal cold hemoglobinuria. Lab Med.

[80] Barcellini W, Fattizzo B. Clinical applications of hemolytic markers in the differential diagnosis and Management of Hemolytic Anemia. Dis Markers. 2015;2015:635670. Available from: 10.1155/2015/635670. 10.1155/2015/635670 PMC4706896 26819490

[81] Audia S, Bach B, Samson M, Lakomy D, Bour J-B, Burlet B (2018). Venous thromboembolic events during warm autoimmune hemolytic anemia. PLoS One.

[82] Randen U, Trøen G, Tierens A, Steen C, Warsame A, Beiske K (2014). Primary cold agglutinin-associated lymphoproliferative disease: a B-cell lymphoma of the bone marrow distinct from lymphoplasmacytic lymphoma. Haematologica..

[83] Fattizzo B, Zaninoni A, Gianelli U, Zanella A, Cortelezzi A, Kulasekararaj AG (2018). Prognostic impact of bone marrow fibrosis and dyserythropoiesis in autoimmune hemolytic anemia. Am J Hematol.

[84] Fattizzo B, Giannotta J, Zaninoni A, Kulasekararaj A, Cro L, Barcellini W. Small Paroxysmal Nocturnal Hemoglobinuria Clones in Autoimmune Hemolytic Anemia: Clinical Implications and Different Cytokine Patterns in Positive and Negative Patients. Front Immunol. 2020;11:1006. Available from: https://www.ncbi.nlm.nih.gov/pmc/articles/PMC7287021/. 10.3389/fimmu.2020.01006 PMC7287021 32582157

[85] Fattizzo B, Michel M, Zaninoni A, Giannotta J, Guillet S, Frederiksen H, et al. Efficacy of recombinant erythropoietin in autoimmune haemolytic anaemia: a multicentre international study. Haematologica, haematol. 2020. Available from: 10.3324/haematol.2020.250522. 10.3324/haematol.2020.250522 PMC7849557 32354865

[86] Berentsen S, Ulvestad E, Gjertsen BT, Hjorth-Hansen H, Langholm R, Knutsen H (2004). Rituximab for primary chronic cold agglutinin disease: a prospective study of 37 courses of therapy in 27 patients. Blood..

[87] Koffas A, Dolman GE, Kennedy PT (2018). Hepatitis B virus reactivation in patients treated with immunosuppressive drugs: a practical guide for clinicians. Clin Med (Lond).

[88] Lechner K, Jäger U (2010). How I treat autoimmune hemolytic anemias in adults. Blood..

[89] Crowther M, Chan YLT, Garbett IK, Lim W, Vickers MA, Crowther MA (2011). Evidence-based focused review of the treatment of idiopathic warm immune hemolytic anemia in adults. Blood..

[90] Michel M, Terriou L, Roudot-Thoraval F, Hamidou M, Ebbo M, Le Guenno G (2017). A randomized and double-blind controlled trial evaluating the safety and efficacy of rituximab for warm auto-immune hemolytic anemia in adults (the RAIHA study). Am J Hematol.

[91] Birgens H, Frederiksen H, Hasselbalch HC, Rasmussen IH, Nielsen OJ, Kjeldsen L (2013). A phase III randomized trial comparing glucocorticoid monotherapy versus glucocorticoid and rituximab in patients with autoimmune haemolytic anaemia. Br J Haematol.

[92] Chao S-H, Chang Y-L, Yen J-C, Liao H-T, Wu T-H, Yu C-L (2020). Efficacy and safety of rituximab in autoimmune and microangiopathic hemolytic anemia: a systematic review and meta-analysis. Exp Hematol Oncol.

[93] Barcellini W, Zaja F, Zaninoni A, Imperiali FG, Battista ML, Di Bona E (2012). Low-dose rituximab in adult patients with idiopathic autoimmune hemolytic anemia: clinical efficacy and biologic studies. Blood..

[94] Reynaud Q, Durieu I, Dutertre M, Ledochowski S, Durupt S, Michallet A-S (2015). Efficacy and safety of rituximab in auto-immune hemolytic anemia: a meta-analysis of 21 studies. Autoimmun Rev.

[95] Fattizzo B, Zaninoni A, Pettine L, Cavallaro F, Di Bona E, Barcellini W (2019). Low-dose rituximab in autoimmune hemolytic anemia: 10 years after. Blood..

[96] Laribi K, Bolle D, Ghnaya H, Sandu A, Besançon A, Denizon N (2016). Rituximab is an effective and safe treatment of relapse in elderly patients with resistant warm AIHA. Ann Hematol.

[97] Coon WW (1985). Splenectomy in the treatment of hemolytic anemia. Arch Surg.

[98] Patel NY, Chilsen AM, Mathiason MA, Kallies KJ, Bottner WA (2012). Outcomes and complications after splenectomy for hematologic disorders. Am J Surg.

[99] Sys J, Provan D, Schauwvlieghe A, Vanderschueren S, Dierickx D (2017). The role of splenectomy in autoimmune hematological disorders: outdated or still worth considering?. Blood Rev.

[100] Kaufman M, Limaye SA, Driscoll N, Johnson C, Caramanica A, Lebowicz Y (2009). A combination of rituximab, cyclophosphamide and dexamethasone effectively treats immune cytopenias of chronic lymphocytic leukemia. Leuk Lymphoma.

[101] Schöllkopf C, Kjeldsen L, Bjerrum OW, Mourits-Andersen HT, Nielsen JL, Christensen BE (2006). Rituximab in chronic cold agglutinin disease: a prospective study of 20 patients. Leuk Lymphoma.

[102] Berentsen S, Randen U, Oksman M, Birgens H, Tvedt THA, Dalgaard J (2017). Bendamustine plus rituximab for chronic cold agglutinin disease: results of a Nordic prospective multicenter trial. Blood.

[103] Berentsen S, Randen U, Vågan AM, Hjorth-Hansen H, Vik A, Dalgaard J (2010). High response rate and durable remissions following fludarabine and rituximab combination therapy for chronic cold agglutinin disease. Blood..

[104] Röth A, Bommer M, Hüttmann A, Herich-Terhürne D, Kuklik N, Rekowski J (2018). Eculizumab in cold agglutinin disease (DECADE): an open-label, prospective, bicentric, nonrandomized phase 2 trial. Blood Adv.

[105] Makishima K, Obara N, Ishitsuka K, Sukegawa S, Suma S, Kiyoki Y (2019). High efficacy of eculizumab treatment for fulminant hemolytic anemia in primary cold agglutinin disease. Ann Hematol.

[106] Neave L, Wilson AJ, Lissack M, Scully M. Severe refractory idiopathic warm autoimmune haemolytic anaemia responsive to complement inhibition with eculizumab. BMJ Case Rep. 2018;11(1):e226429. 10.1136/bcr-2018-226429 PMC6301595 30567234

[107] Schwartz J, Padmanabhan A, Aqui N, Balogun RA, Connelly-Smith L, Delaney M (2016). Guidelines on the use of therapeutic apheresis in clinical practice-evidence-based approach from the writing Committee of the American Society for apheresis: the seventh special issue. J Clin Apher.

[108] Michalak SS. Autoimmune hemolytic Anemia. In: Gu D, Dupre ME, editors. Encyclopedia of gerontology and population aging. Cham: Springer International Publishing; 2019. 1–10. Available from: doi: 10.1007/978-3-319-69892-2_62-1.

[109] Valentini RP, Imam A, Warrier I, Ellis D, Ritchey AK, Ravindranath Y (2006). Sirolimus rescue for tacrolimus-associated post-transplant autoimmune hemolytic anemia. Pediatr Transplant.

[110] Miano M, Calvillo M, Palmisani E, Fioredda F, Micalizzi C, Svahn J (2014). Sirolimus for the treatment of multi-resistant autoimmune haemolytic anaemia in children. Br J Haematol.

[111] Ratnasingam S, Walker PA, Tran H, Kaplan ZS, McFadyen JD, Tran H (2016). Bortezomib-based antibody depletion for refractory autoimmune hematological diseases. Blood Adv.

[112] Fadlallah J, Michel M, Crickx E, Limal N, Costedoat N, Malphettes M (2019). Bortezomib and dexamethasone, an original approach for treating multi-refractory warm autoimmune haemolytic anaemia. Br J Haematol.

[113] Even-Or E, Naser Eddin A, Shadur B, Dinur Schejter Y, Najajreh M, Zelig O (2020). Successful treatment with daratumumab for post-HSCT refractory hemolytic anemia. Pediatr Blood Cancer.

[114] Schuetz C, Hoenig M, Moshous D, Weinstock C, Castelle M, Bendavid M (2018). Daratumumab in life-threatening autoimmune hemolytic anemia following hematopoietic stem cell transplantation. Blood Adv.

[115] Byrd JC, Wierda WG, Schuh A, Devereux S, Chaves JM, Brown JR, et al. Acalabrutinib monotherapy in patients with relapsed/refractory chronic lymphocytic leukemia: updated phase 2 results. Blood. 2019;26.

[116] Nader K, Patel M, Ferber A (2013). Ofatumumab in rituximab-refractory autoimmune hemolytic anemia associated with chronic lymphocytic leukemia: a case report and review of literature. Clin Lymphoma Myeloma Leuk.

[117] Gómez-Almaguer D, Solano-Genesta M, Tarín-Arzaga L, Herrera-Garza JL, Cantú-Rodríguez OG, Gutiérrez-Aguirre CH (2010). Low-dose rituximab and alemtuzumab combination therapy for patients with steroid-refractory autoimmune cytopenias. Blood..

[118] Blumberg LJ, Humphries JE, Jones SD, Pearce LB, Holgate R, Hearn A (2019). Blocking FcRn in humans reduces circulating IgG levels and inhibits IgG immune complex-mediated immune responses. Sci Adv.

[119] Sharp JA, Whitley PH, Cunnion KM, Krishna NK (2014). Peptide inhibitor of complement C1, a novel suppressor of classical pathway activation: mechanistic studies and clinical potential. Front Immunol.

[120] Shi J, Rose EL, Singh A, Hussain S, Stagliano NE, Parry GC (2014). TNT003, an inhibitor of the serine protease C1s, prevents complement activation induced by cold agglutinins. Blood..

[121] Wouters D, Stephan F, Strengers P, de Haas M, Brouwer C, Hagenbeek A (2013). C1-esterase inhibitor concentrate rescues erythrocytes from complement-mediated destruction in autoimmune hemolytic anemia. Blood..

[122] Jäger U, D’Sa S, Schörgenhofer C, Bartko J, Derhaschnig U, Sillaber C, et al. Inhibition of complement C1s improves severe hemolytic anemia in cold agglutinin disease: a first-in-human trial. Blood. 2019;133(9):893–901. 10.1182/blood-2018-06-856930 PMC6396179 30559259

[123] Gelbenegger G, Schoergenhofer C, Derhaschnig U, Buchtele N, Sillaber C, Fillitz M (2020). Inhibition of complement C1s in patients with cold agglutinin disease: lessons learned from a named patient program. Blood Adv.

[124] Gertz MA, Qiu H, Kendall L, Saltarelli M, Yednock T, Sankaranarayanan S (2016). ANX005, an inhibitory antibody against C1q, blocks complement activation triggered by cold agglutinins in human disease. Blood..

[125] Risitano AM, Notaro R, Marando L, Serio B, Ranaldi D, Seneca E (2009). Complement fraction 3 binding on erythrocytes as additional mechanism of disease in paroxysmal nocturnal hemoglobinuria patients treated by eculizumab. Blood..

[126] Mastellos DC, Yancopoulou D, Kokkinos P, Huber-Lang M, Hajishengallis G, Biglarnia AR (2015). Compstatin: a C3-targeted complement inhibitor reaching its prime for bedside intervention. Eur J Clin Investig.

[127] Baas I, Delvasto-Nuñez L, Ligthart P, Brouwer C, Folman C, Reis ES (2020). Complement C3 inhibition by compstatin Cp40 prevents intra- and extravascular hemolysis of red blood cells. Haematologica..

[128] Szilágyi K, Hajdú I, Flachner B, Lőrincz Z, Balczer J, Gál P, et al. Design and selection of novel C1s inhibitors by in Silico and *in vitro* approaches. Molecules. 2019;24(20):3641. 10.3390/molecules24203641 PMC6832932 31600984

